# Effect of post-exercise lactate administration on glycogen repletion and signaling activation in different types of mouse skeletal muscle

**DOI:** 10.1016/j.crphys.2020.07.002

**Published:** 2020-07-30

**Authors:** Kenya Takahashi, Yu Kitaoka, Yutaka Matsunaga, Hideo Hatta

**Affiliations:** a Department of Sports Sciences, The University of Tokyo, 3-8-1, Komaba, Meguro-ku, Tokyo, 153-8902, Japan; b Department of Human Sciences, Kanagawa University, 3-27-1 Rokkakubashi, Kanagawa-ku, Yokohama, Kanagawa, 221-8686, Japan

**Keywords:** Lactate, Glycogen, Recovery, Skeletal muscle, Exercise, Glyconeogenesis

## Abstract

Lactate is not merely a metabolic intermediate that serves as an oxidizable and glyconeogenic substrate, but it is also a potential signaling molecule. The objectives of this study were to investigate whether lactate administration enhances post-exercise glycogen repletion in association with cellular signaling activation in different types of skeletal muscle. Eight-week-old male ICR mice performed treadmill running (20 m/min for 60 min) following overnight fasting (16 h). Immediately after the exercise, animals received an intraperitoneal injection of phosphate-buffered saline or sodium lactate (equivalent to 1 g/kg body weight), followed by oral ingestion of water or glucose (2 g/kg body weight). At 60 min of recovery, glucose ingestion enhanced glycogen content in the soleus, plantaris, and gastrocnemius muscles. In addition, lactate injection additively increased glycogen content in the plantaris and gastrocnemius muscles, but not in the soleus muscle. Nevertheless, lactate administration did not significantly alter protein levels related to glucose uptake and oxidation in the plantaris muscle, but enhanced phosphorylation of TBC1D1, a distal protein regulating GLUT4 translocation, was observed in the soleus muscle. Muscle FBP2 protein content was significantly higher in the plantaris and gastrocnemius muscles than in the soleus muscle, whereas MCT1 protein content was significantly higher in the soleus muscle than in the plantaris and gastrocnemius muscles. The current findings suggest that an elevated blood lactate concentration and post-exercise glucose ingestion additively enhance glycogen recovery in glycolytic phenotype muscles. This appears to be associated with glyconeogenic protein content, but not with enhanced glucose uptake, attenuated glucose oxidation, or lactate transport protein.

## Highlights

• Lactate is an oxidizable and glyconeogenic metabolic intermediate and signaling molecule.
• Lactate injection and glucose ingestion additively enhanced post-exercise glycogen recovery in plantaris and gastrocnemius, but not soleus muscles.
• Lactate injection did not change glucose uptake and oxidation in plantaris muscle, but it enhanced phosphorylation of TBC1D1 in soleus muscle.
• Muscle protein isotype FBP2 content was significantly higher in plantaris and gastrocnemius muscles than in soleus muscle.
• The current findings suggest that elevated circulating lactate and carbohydrate consumption additively enhances post-exercise glycogen recovery in glycolytic phenotype muscles in association with glyconeogenic enzyme protein.

## Graphical abstract

**Figure:**
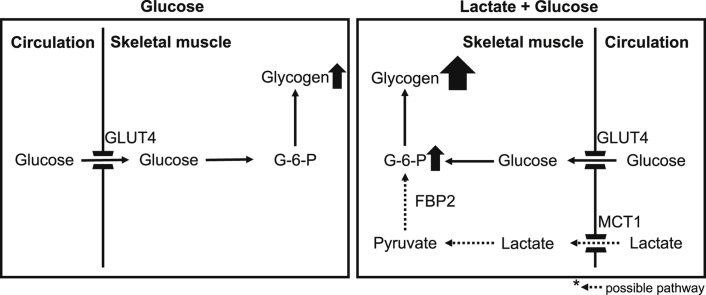

## 1 Introduction

Glycogen is formed as a branched polymer of glucose serving as an essential energy deposit stored predominantly in skeletal muscle. Given the determinant role of muscle glycogen in exercise physiology (Hawley et al. 1997; Watanabe & Wada 2019; Jensen et al. 2020; Chin & Allen 1997; Bergström et al. 1967; Alghannam et al. 2016; Fallowfield et al. 1995; Burke et al., 2017a, Burke et al., 2017b), glycogen replenishment is one of the top priorities for people who participate in multiple and successive athletic events (Burke et al., 2017a, Burke et al., 2017b; Peake 2019). Carbohydrate intake after exercise is, therefore, recommended for glycogen resynthesis according to sports nutrition guidelines (Thomas et al. 2016; Kerksick et al. 2018). Although circulating metabolite concentrations can vary depending on the intensity of exercise, the impact of interaction between consumed carbohydrates and circulating metabolites on post-exercise glycogen restoration is not fully understood.

Lactate had long been considered to be a waste product that is produced in skeletal muscle and accumulated in the circulation as exercise intensity increases (Gladden 2004). Although serving as a triose precursor for hepatic gluconeogenesis via the Cori cycle was thought to be the sole fate of lactate (Cori & Cori 1929), it is now widely accepted that oxidation is the major pathway of lactate (Mazzeo et al. 1986). Moreover, previous studies have provided evidence that skeletal muscle possesses enzymes that catalyze lactate to glycogen (Opie & Newsholme 1967; McLane & Holloszy 1979; Crabtree et al. 1972), and that lactate is incorporated into glycogen in skeletal muscle (McLane & Holloszy 1979; Shiota et al. 1984; Jin et al. 2015; Bonen et al. 1990). Intriguingly, there is growing evidence that lactate acts as a signaling molecule that activates cellular signaling (Cai et al. 2008; Liu et al. 2009) and induces metabolic adaptation (Brooks 2018; Hashimoto et al. 2007). A previous study reported that lactate infusion enhanced the disappearance of 2-deoxy-glucose (2-DG), a glucose analog, from blood circulation in rats (Pagano et al. 1997). Another study reported muscle phenotype-dependent signaling activation after lactate administration in mice (Cerda-Kohler et al. 2018). These findings led us to hypothesize that lactate administration alters signaling pathways associated with glucose uptake and oxidation depending on muscle phenotype, leading to enhanced glycogen recovery.

In this study, we first assessed post-exercise lactate injection with or without glucose ingestion on blood substrate concentrations. Secondly, we examined whether post-exercise lactate administration and glucose ingestion additively enhance glycogen content in skeletal muscle. Next, we investigated protein levels associated with glucose uptake and oxidation. Lastly, we evaluated muscle phenotype differences for key proteins regulating gluconeogenesis from circulating lactate. We analyzed an oxidative phenotype muscle (soleus muscle), a major site of lactate oxidation, and glycolytic phenotype muscles (plantaris and gastrocnemius muscles), primary sites of lactate production (Dimmer et al. 2000; Wilson et al. 1998; Bonen et al. 2000), because their differences in lactate metabolism could result in different outcomes.

## 2 Methods

### 2.1 Animals

Male ICR mice (8 weeks old; CLEA Japan, Tokyo, Japan) were used throughout this study. Mice were individually housed on a 12:12-h light–dark cycle (dark 7:00 AM to 7:00 PM) in an air-conditioned room at 22 °C. All mice were provided with standard chow (MF, Oriental Yeast, Tokyo, Japan) and water ad libitum during the experimental periods. All experiments were approved by the Animal Experimental Committee of The University of Tokyo (approval number 27-14).

### 2.2 Experimental procedure

#### 2.2.1 Experiment 1: post-exercise substrate levels in the circulation after lactate administration

**Fig. 1:**
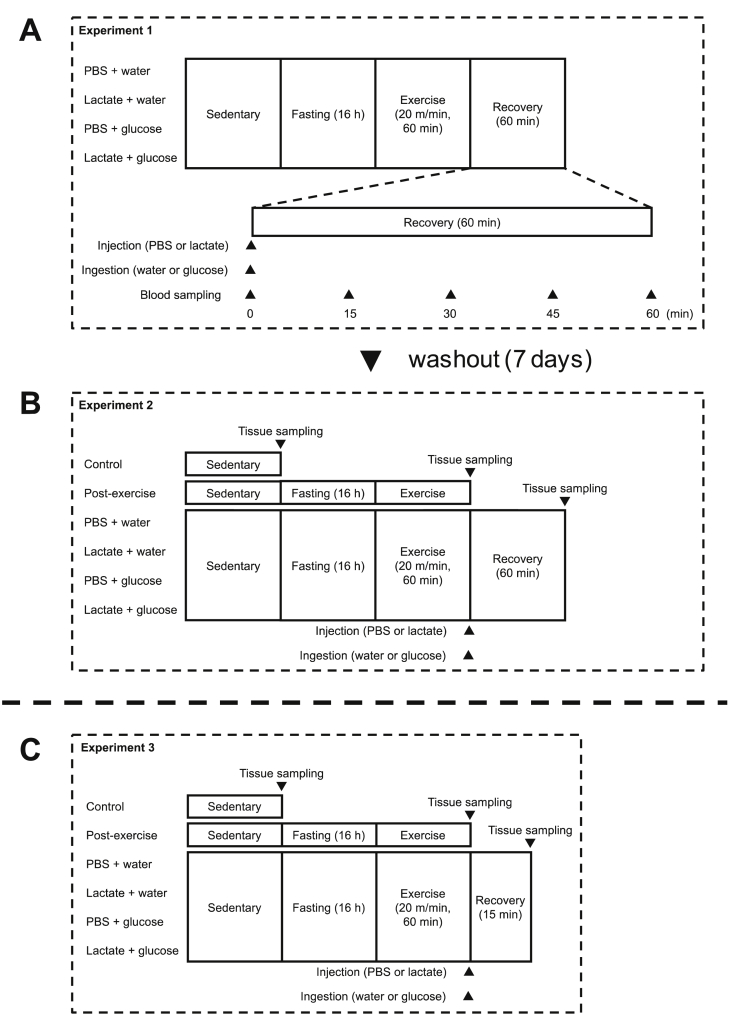
Experimental procedure. Experiment 1: blood and plasma substrate concentrations were taken before and after the intraperitoneal injection of PBS or lactate, followed by the ingestion of water or glucose (A). Experiment 2: soleus, plantaris, and gastrocnemius muscles were taken after 60 min of recovery period for the determination of glycogen content (B). Experiment 3: soleus and plantaris muscles were taken 15 min after PBS or lactate administration for the evaluation of proteins associated with glucose uptake and oxidation (C).

Figure 1 shows a schematic overview of the experiments. Animals were randomly allocated to one of four groups as follows: a phosphate-buffered saline (PBS) ​+ ​water administration group (PW, n ​= ​8), a lactate ​+ ​water administration group (LW, n ​= ​8), a PBS ​+ ​glucose administration group (PG, n ​= ​8), and a lactate ​+ ​glucose administration group (LG, n ​= ​8). Before the day of the experiment, all animals were acclimated to running on a treadmill (MK-680; Muromachi Kikai Co., Inc., Tokyo, Japan) at a speed of 20 ​m/min for 5 ​min for 3 days. Following overnight fasting for 16 ​h from 7:00 PM to 11:00 AM, the animals performed treadmill running at a speed of 20 ​m/min for 60 ​min. The duration of fasting and exercise was set according to a previous study (Matsunaga et al. 2018). Immediately after the exercise, animals received an intraperitoneal injection of PBS or sodium lactate [equivalent to 1 g/kg body weight (BW), 200 mg/mL concentration], followed by an ingestion of distilled water or glucose (2 g/kg BW, 200 mg/mL concentration) via oral gavage. The glucose volume is expected to maximize glycogen resynthesis from glucose (Betts & Williams 2010). Before and after administration (0, 15, 30, 45, and 60 min), tail vein blood samples were taken using heparinized tubes, and were centrifuged (3,000 g, 10 min, 4 °C) to collect plasma samples. During the recovery period, food and water was withheld.

#### 2.2.2 Experiment 2: post-exercise glycogen recovery after lactate administration

Following a washout period of 7 days, animals were treated in the same way as described in Experiment 1 without blood sampling (Fig. 1B). At 60 min after the exercise, tissues were taken, rapidly frozen in liquid nitrogen, and stored at −80 °C until analysis. As a reference, data from a non-fasting control group (CON, n = 8) and a post-exercise group (EXE, n = 8) are shown.

#### 2.2.3 Experiment 3: effects of post-exercise lactate administration on proteins associated with glucose uptake and oxidation at 15 min recovery

Animals were assigned to one of six groups (n = 7 in each group) and were treated in the same way as described above (Fig. 1C). At 15 min after exercise, tissues were taken, rapidly frozen in liquid nitrogen, and stored at −80 °C until analysis.

#### 2.2.4 Experiment 4: lactate transporter and gluconeogenic enzyme in different types of skeletal muscle

To evaluate protein content differences, soleus, plantaris, and gastrocnemius muscles were taken, rapidly frozen in liquid nitrogen, and stored at −80 °C until analysis.

### 2.3 Blood and plasma substrates

Portable analyzers were used for the measurement of blood glucose (GLUCOCARD PlusCare; Arkray, Kyoto, Japan) and lactate (Lactate Pro 2; Arkray). Plasma-free fatty acid (FFA) concentration was measured using a kit (NEFA C test kit; FUJIFILM Wako, Osaka, Japan). Plasma insulin concentration was determined using an ELISA kit for mouse insulin (Morinaga Bioscience Laboratory, Kanagawa, Japan) following the manufacturer's instructions.

### 2.4 Muscle glycogen

Whole soleus, plantaris, and gastrocnemius muscles were heated at 100 °C in 30% (wt/vol) KOH saturated with Na_2_SO_4_ until completely dissolved. The sample solutions were mixed with 99.5% ethanol, and were put on ice for 30 min. After being centrifuged at 10,000 g for 10 min at 4 °C, the sample pellets (glycogen precipitates) were hydrolyzed to glucose in 1 M HCl at 100 °C for 2 h. The sample solutions were neutralized with 1 M NaOH. Glycogen content was determined using a kit (Glucose CII kit; Fujifilm Wako) according to the manufacturer's instructions.

### 2.5 Muscle metabolites

Given that some metabolites exist at low concentration in the skeletal muscle and require a large muscle mass for their measurement, whole gastrocnemius muscle was used for metabolite analysis. Briefly, whole gastrocnemius muscles were homogenized in 0.6 N HClO_4_ buffer using a μT-01 bead crusher (TITEC, Saitama, Japan). After centrifugation at 12,000 g for 10 min at 4 °C, the supernatants were neutralized with 1 M NaOH. Glucose and G-6-P concentrations were measured using enzymatic colorimetric methods described in a previous study with minor modifications (Zhu et al. 2011). In brief, 30 μL of neutralized samples were added to 70 μL of either glucose assay solution [20 μM MgCl_2_, 25 μM NADP^+^, 0.5 mM WST-1, 10 μM 1-mPMS, 0.2 U glucose dehydrogenase, and 50 mM Tris–HCl (pH 8.5)] or G-6-P assay solution [20 μM MgCl_2_, 25 μM NADP^+^, 0.5 mM WST-1, 10 μM 1-mPMS, 0.2 U G-6-P dehydrogenase, and 50 mM Tris–HCl (pH 8.5)]. The absorbance at 440 nm was read after incubation for 30 min at room temperature in the dark. Lactate concentration was measured as described elsewhere (Gutmann 1974; Hoshino et al. 2015). In brief, 50 μL of neutralized sample was mixed with 1,000 μL of lactate assay solution (0.4 M hydrazine, 0.5 M glycine, 0.4 mM NAD^+^, and 1,000 U l-lactate dehydrogenase). After being incubated for 30 min at room temperature, the absorbance at 340 nm was measured.

### 2.6 Western blotting

Whole soleus, plantaris, and gastrocnemius muscles were homogenized using a μT-01 bead crusher (TITEC) diluted 20 times (vol/wt) in an ice-cold radioimmunoprecipitation assay (RIPA) buffer (25 mM Tris–HCl, pH 7.6, 150 mM NaCl, 1% NP-40, and 1% sodium deoxycholate) supplemented with a protease inhibitor mixture (cOmplete Mini, ETDA-free; Roche Applied Science, Indianapolis, IN, USA) and a phosphatase inhibitor mixture (PhosSTOP; Roche Applied Science). The homogenates were rotated on ice for 60 min and centrifuged at 1,500 g at 4 °C for 20 min. The total protein content of the samples was determined using a BCA protein assay (TaKaRa BIO INC., Shiga, Japan). Equal amounts of protein, depending on the protein of interest, were loaded onto sodium dodecyl sulfate-polyacrylamide gels (7.5% or 10%) and separated by electrophoresis. Proteins were transferred to polyvinylidene difluoride membranes, and western blotting was carried out according to the protocol described in our previous studies (Takahashi et al. 2019; Takahashi et al. 2020). The primary and secondary antibodies used in the present study are described below. Blots were scanned and quantified using ChemiDoc XRS (Bio-Rad Laboratories, Hercules, CA, USA) and Quantity One (version 4.5.2, Bio-Rad). Ponceau staining was used to verify consistent loading.

### 2.7 Antibodies for western blotting

Primary antibodies used in the present study are as follows: phosphorylated (p-) RAC-alpha serine/threonine-protein kinase (Akt)^Thr308^ [#9275; Cell Signaling Technology (CST) Japan, Tokyo, Japan]; p-Akt^Ser473^ (#9271; CST Japan); Akt (#9272; CST Japan); p-mammalian or mechanistic target of rapamycin (mTOR)^Ser2448^ (#2481; CST Japan); mTOR (#2983; CST Japan); p-ribosomal protein S6 kinase beta-1 (p70S6K)^Thr389^ (#9205; CST Japan); p70S6K (#9202; CST Japan); p-5′ adenosine monophosphate-activated protein kinase (AMPK)^Thr172^ (#2513; CST Japan); AMPK (#2532; CST Japan); p-acetyl-CoA carboxylase (ACC)^Ser79^ (#3661; CST Japan); ACC (#3662; CST Japan); p-pyruvate dehydrogenase (PDH)^Ser293^ (#ab177461; Abcam, Cambridge, UK); PDH (#ab168379; Abcam, Cambridge, UK); PDH kinase 4 (PDK4) (#ab214938; Abcam); p-glycogen synthase (GS)^Ser641^ (#3891; CST Japan); GS (#3893; CST Japan); p-Akt substrate of 160 kDa (AS160)^Thr642^ (#8881; CST Japan); p-AS160^Ser588^ (#8730; CST Japan); AS160 (#2670; CST Japan); p-tre-2/USP6, BUB2, cdc16 domain family member 1 (TBC1D1)^Ser231^ (#07-2268; MilliporeSigma, Bedford, MA, USA); TBC1D1 (#2670; CST Japan); and fructose-1,6-bisphosphatase 2 (FBP2) (#ab131253; Abcam). Monocarboxylate transporter 1 (MCT1) antibody was raised in rabbits against the C-terminal region of the MCT1 (Qiagen, Japan), as used in previous studies (Takahashi et al. 2019; Takahashi et al. 2020; Hoshino et al. 2014; Kitaoka et al. 2014). The following secondary antibodies were used in the current study: rabbit anti-goat IgG (H&L) (#A106PU; American Qualex, San Clemente, CA, USA) and mouse anti-goat IgG (H&L) (#A102PT; American Qualex).

### 2.8 Statistical analysis

All data are expressed as mean ± standard error of the mean (SEM). For the analysis of time course changes in circulating substrate concentrations, the Turkey-Kramer multiple-comparison test was performed at each time point. To determine the differences among the four groups, two-way analysis of variance (ANOVA; lactate × glucose) was performed. When an interaction between the two factors was found to be significant, Turkey-Kramer multiple comparison test was performed. To detect the differences among skeletal muscles (soleus, plantaris, and gastrocnemius muscles), one-way ANOVA was performed, followed by the Turkey-Kramer multiple-comparison test. All statistical analyses were performed by GraphPad Prism (Ver. 7.0, Macintosh, GraphPad Software, La Jolla, CA, USA). For all statistical evaluations, p < 0.05 was considered to be significant. To minimize the chances of committing type II errors, results within the range 0.05 ≤ p ≤ 0.1 are shown as tendencies.

## 3 Results

### 3.1 Experiment 1: post-exercise substrate levels in the circulation after lactate administration

**Fig. 2:**
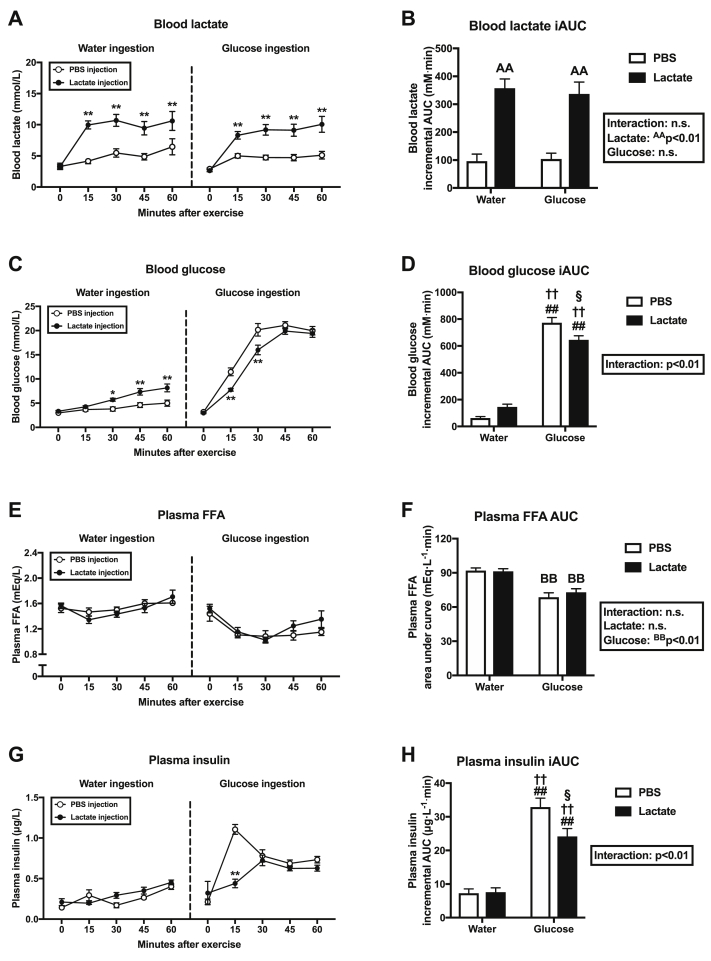
The left panels show blood lactate (A), blood glucose (C), plasma FFA (E), and plasma insulin (G) concentrations during 60 ​min of recovery (Experiment 1). The right panels show iAUC measurements of lactate (B), glucose (D), and insulin (H) and AUC measurement of FFA (F). Data are expressed as means ​± ​SEM with n ​= ​7–8. ∗p ​< ​0.05, ∗∗p ​< ​0.01: significant difference between the two groups at each time point. n.s.: not significant. ^AA^p<0.01: main effect of lactate injection. ^BB^p<0.01: main effect of glucose ingestion. ^##^p < 0.01 vs. PW group; ^††^p < 0.01 vs. LW group; ^§^p < 0.05 vs. PG group.

Blood lactate concentration was significantly more elevated in the LW and LG groups than in the PW and PG groups at 15, 30, 45, and 60 min of recovery (Fig. 2A), resulting in a main positive effect of lactate injection on the incremental area under the curve (iAUC) of blood lactate (Fig. 2B). Blood glucose level was significantly higher in the LW group than in the PW group at 30, 45, 60 min of recovery (Fig. 2C), and significantly higher in the PG group than in the LG group at 15 and 30 min of recovery (Fig. 2C). Blood glucose iAUC was significantly higher in the PG and the LG groups than in the PW and LW groups, and significantly higher in the PG group than in the LG group. Glucose ingestion had a major effect on plasma FFA AUC (Fig. 2F). The plasma insulin level did not differ between the PW and LW groups (Fig. 2G), but it was significantly lower in the LG group than in the PG group at 15 min of recovery (Fig. 2G). Plasma insulin iAUC was significantly higher in the PG and the LG groups than in the PW and LW groups, and significantly higher in the PG group than in the LG group (Fig. 2H).

### 3.2 Experiment 2: post-exercise glycogen recovery after lactate administration

**Fig. 3:**
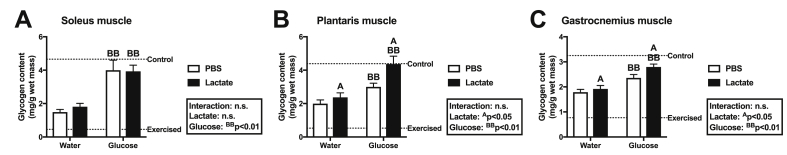
Glycogen concentration in the soleus muscle (A), plantaris muscle (B), and gastrocnemius muscle (C) after 60 min of recovery (Experiment 2). Data are expressed as means ± SEM with n = 7–8. n.s.: not significant. ^A^p<0.05: main effect of lactate injection. ^BB^p<0.01: main effect of glucose ingestion.

**Fig. 4:**
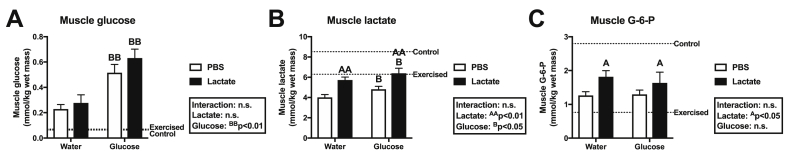
Concentrations of glucose (A), lactate (B), and G-6-P (C) in the gastrocnemius muscle after 60 min of recovery (Experiment 2). Data are expressed as means ± SEM with n = 7–8. n.s.: not significant. ^A^p<0.05, ^AA^p<0.01: main effect of lactate injection. ^B^p < 0.05, ^BB^p<0.01: main effect of glucose ingestion.

**Fig. 5:**
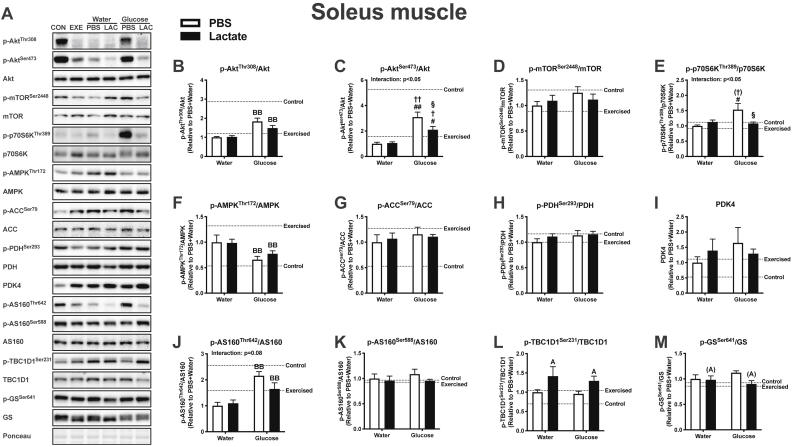
Protein content of the soleus muscle at 15 min of recovery (Experiment 3). Data are expressed as means ± SEM with n = 6–7. ^(A)^p ≤ 0.10, ^A^p<0.05: main effect of lactate injection. ^BB^p<0.01: main effect of glucose ingestion. ^#^p < 0.05, ^##^p < 0.01 vs. PW group; ^†^p ≤ 0.10, ^†^p < 0.05, ^††^p < 0.01 vs. LW group; ^§^p < 0.05 vs. PG group.

**Fig. 6:**
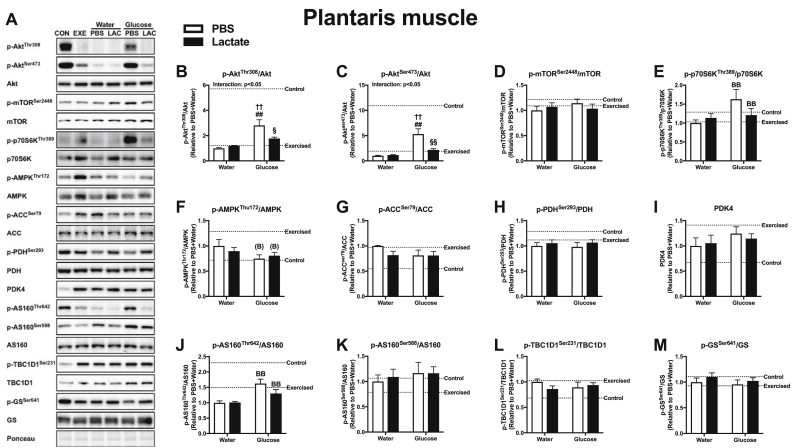
Protein content of the plantaris muscle at 15 min of recovery period (Experiment 3). Data are expressed as means ± SEM with n = 5–7. ^(B)^p ≤ 0.10, ^BB^p<0.01: main effect of glucose ingestion. ^##^p < 0.01 vs. PW group; ^††^p < 0.01 vs. LW group; ^§^p < 0.05, ^§§^p < 0.01 vs. PG group.

To clarify whether post-exercise lactate administration additively enhances glycogen repletion, we analyzed muscle glycogen concentration. At 60 min of recovery, we observed a positive major effect of glucose ingestion on glycogen content in the soleus muscle (Fig. 3A), plantaris muscle (Fig. 3B), and gastrocnemius muscle (Fig. 3C). Although lactate injection had no effect on glycogen content in the soleus muscle (Fig. 3A), we found a positive main effect of lactate injection on glycogen concentration in the plantaris muscle (Fig. 3B) and the gastrocnemius muscle (Fig. 3C). To seek the potential mechanisms underlying enhanced glycogen in the plantaris and gastrocnemius muscles, we next analyzed metabolite levels in the gastrocnemius muscle. Glucose ingestion had a major positive effect on levels of glucose (Fig. 4A) and lactate (Fig. 4B) in the gastrocnemius muscle. In addition, another effect of lactate injection was observed in lactate (Fig. 4B) and G-6-P (Fig. 4C) concentrations in the gastrocnemius muscle.

### 3.3 Experiment 3: effects of post-exercise lactate administration on proteins associated with glucose uptake and oxidation at 15 min recovery

Lactate is recognized as a molecule that activates cellular signaling. To assess whether differences in glycogen content at 60 min recovery could be explained by differences in glucose uptake and oxidation, we analyzed the phosphorylation status of proteins at 15 min recovery, when circulating glucose and insulin levels were significantly lower in the LG group than in the WG group. In this measurement, we used the soleus and plantaris muscles, but not the gastrocnemius muscle, because we observed a similar glycogen result in the plantaris and gastrocnemius muscles. In the soleus muscle, glucose ingestion was observed to have a large effect in p-Akt^Thr308^ (Fig. 5B), p-AMPK^Thr172^ (Fig. 5F), and p-AS160^Thr642^ (Fig. 5J). In addition, p-Akt^Ser473^ was significantly higher in the PG and LG groups than in the PW and LW groups, and higher in the PG group than in the LG group (Fig. 5C). The value for p-p70S6K was significantly higher in the PG group than in the PW and LG groups (Fig. 5E). Moreover, a positive main effect of lactate administration was found in p-TBC1D1^Ser231^ (Fig. 5L). In the plantaris muscle, a main effect of glucose ingestion was observed in p-p70S6K^Thr389^ (Fig. 6E) and p-AS160^Thr642^ (Fig. 6J). Both p-Akt^Thr308^ (Fig. 6B) and p-Akt^Ser473^ (Fig. 6C) were significantly higher in the PG group than in the PW, LW, and LG groups. No significant main effect of lactate or glucose was found in mTOR^Ser2448^, ACC^Ser79^, PDH^Ser293^, PDK4, AS160^Ser588^, and GS^Ser641^ in the soleus (Fig. 5) and plantaris muscles (Fig. 6), and in AMPK^Thr172^ in the plantaris muscle (Fig. 6F).

### 3.4 Experiment 4: lactate transporter and gluconeogenic enzyme in different types of skeletal muscle

**Fig. 7:**
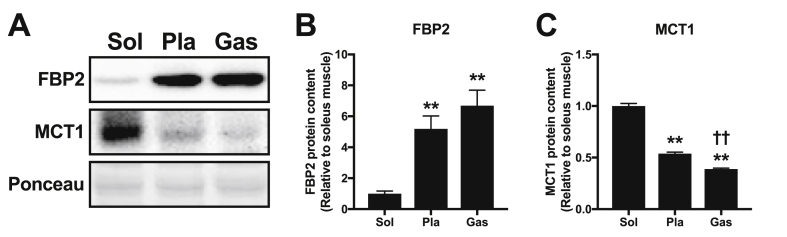
Representative Western blot results (A) and content of proteins FBP2 (B) and MCT1 (C) in the soleus, plantaris, and gastrocnemius muscles (Experiment 4). Data are expressed as means ​± ​SEM with n ​= ​7. ∗∗p ​< ​0.01 vs. soleus muscle. ^††^p < 0.01 vs. plantaris muscle.

To further explore the mechanisms underlying muscle phenotype-dependent glycogen recovery after lactate administration, we measured the content of the FBP2 protein, the muscle isotype of FBP that catalyzes glycogen synthesis from lactate, and MCT1, a lactate transporter that facilitates lactate uptake into skeletal muscle. Muscle FBP2 protein content was significantly higher in the plantaris and gastrocnemius muscles than in the soleus muscle (Fig. 7B). MCT1 protein content was significantly higher in the soleus muscle than in the plantaris and gastrocnemius muscles (Fig. 7C), and significantly higher in the plantaris muscle than in the gastrocnemius muscle (Fig. 7C).

## 4 Discussion

Lactate is a metabolic intermediate that serves as an oxidizable and glyconeogenic substrate that accumulates in the circulating blood as exercise intensity increases (Gladden 2004). In recent years, lactate has come to be recognized as a signaling molecule (Brooks 2018; Brooks 2020). However, whether an elevated circulating lactate and carbohydrate intake additively enhance post-exercise glycogen recovery, as well as whether lactate alters cellular signaling associated with glucose uptake and oxidation, remained to be fully elucidated. In the present study, we demonstrated that lactate injection and glucose ingestion additively enhanced glycogen concentration in the plantaris and gastrocnemius muscles at 60 min of recovery. Both plantaris and gastrocnemius muscles are composed of more than 90% type II fibers, and they possess higher glycolytic capacity and lower oxidative capacity compared with soleus muscles, which consist of approximately 40% type I fibers (Denies et al. 2014; Augusto et al. 2017; Suzuki 2019). These results suggest that an elevated circulating lactate and carbohydrate intake additively enhance post-exercise glycogen repletion in glycolytic phenotype muscle.

Previous studies reported that co-ingestion of glucose and fructose significantly enhanced blood lactate level (Lecoultre et al. 2010; Rosset et al. 2017); however, in the present study, effect of glucose ingestion was not statistically significant (p = 0.16). After intestinal absorption, glucose can pass through the liver, whereas fructose is converted in the liver to glucose and lactate, which then enters the systemic circulation (Tappy & Rosset 2017; Tappy 2018). We assume that the difference between glucose and fructose resulted in no significant effect of glucose ingestion on blood lactate level in our present study.

Several potential mechanisms contribute to the enhanced glycogen concentration after lactate administration: enhanced glucose uptake, attenuated glucose oxidation, and glyconeogenesis from lactate. We first addressed whether lactate administration enhanced glucose uptake into skeletal muscle. It is well established that lactate is the primary precursor for gluconeogenesis in the liver (Cori & Cori 1929; Bergman et al. 2000; Emhoff et al. 2013; Meyer et al. 2002; Stanley et al. 1988; Donovan & Brooks 1983; Turcotte & Brooks 1990). In accordance with this, blood glucose concentrations at 30, 45, and 60 min were significantly higher in the LW group than in the PW group, raising the possibility that the elevated blood glucose concentration augmented muscle glycogen content in plantaris and gastrocnemius muscles, due to the fact that glucose uptake depends on concentration gradient and that glycogen synthesis depends on the rate of glucose uptake (Hunter et al. 2011). However, because lactate administration did not increase glycogen content in the soleus muscle, we assume that the enhanced glycogen content in the plantaris and gastrocnemius muscles is not primarily due to the elevated blood glucose concentration.

In the current study, blood glucose levels at 15 and 30 min, as well as blood glucose iAUC, were significantly lower in the LG group than in the PG group. A previous study reported that lactate infusion enhanced 2-DG removal from the circulation and uptake of 2-DG into skeletal muscle compared with bicarbonate infusion (Pagano et al. 1997), which suggests that lactate can facilitate glucose uptake into skeletal muscle. To assess whether the lower blood glucose level and the enhanced glycogen content in the plantaris and gastrocnemius muscles after lactate administration could be explained by the differences in glucose uptake facilitated by glucose transporter (GLUT) 4 exocytosis, we measured phosphorylation status of AS160 (also known as TBC1D4) and TBC1D1, distal proteins regulating translocation of GLUT4 to the plasmalemma (Cartee 2015), at 15 min recovery, when circulating glucose and insulin levels were significantly lower in the LG group than in the PG group. In the present study, we found that lactate administration had no effect on AS160^Thr642^, AS160^Ser588^, and TBC1D1^Ser231^ in plantaris muscles. This result may suggest that the lower blood glucose level and enhanced glycogen content in the plantaris and gastrocnemius muscles after lactate injection are not caused by a greater rate of glucose uptake into skeletal muscle. We assume that lactate injection delayed digestion and absorption of ingested glucose, resulting in a lower glucose level.

To the best of our knowledge, the current observation that lactate injection significantly increased p-TBC1D1^Ser231^ in the soleus muscle is a new finding. Although this phosphosite is downstream of AMPK (Taylor et al. 2008), we observed no significant effect of lactate administration on p-AMPK^Thr172^ or p-ACC^Ser79^, indicators of AMPK activity. Previous studies have reported that lactate activates an intracellular signaling pathway by binding to G protein-coupled receptor 81 (Ohno et al. 2018), a lactate-specific receptor (Cai et al. 2008; Liu et al. 2009). These findings suggest that p-TBC1D1^Ser231^ is possibly regulated by another mechanism through lactate signaling in a muscle phenotype-dependent manner. Regardless, lactate administration did not enhance glycogen content in the soleus muscle at 60 min of recovery. Previous studies showed that TBC1D1 protein abundance in mouse soleus muscle was lower than that in the plantaris and gastrocnemius muscles (Taylor et al. 2008), and that soleus glucose uptake during a euglycemic–hyperinsulinemic clamp was unaltered for TBC1D1-deficient mice (Szekeres et al. 2012), suggesting that increased p-TBC1D1^Ser231^ does not necessarily enhance glucose uptake and, therefore, glycogen accumulation in the soleus muscle.

Activation of GS is also a key factor in glycogen synthesis. In the current study, lactate administration did not significantly alter p-GS^Ser641^ in the plantaris muscle, but it significantly elevated the G-6-P concentration in the gastrocnemius muscle. Given that GS is activated not only by dephosphorylation, but also by allosteric stimulator G-6-P (Bouskila et al. 2010; Jensen & Lai 2009), GS activation through the elevation of G-6-P concentration after lactate administration might contribute, in part, to an enhanced glycogen content in the plantaris and gastrocnemius muscles.

PDH is a gateway enzyme permitting the entry of pyruvate derived from carbohydrates into the mitochondrial tricarboxylic acid cycle for oxidation. Given that glycogen accumulation depends not only on glucose uptake, but also on glucose oxidation, PDH activity affects glycogen synthesis (Pilegaard & Neufer 2004; Madar 1989). A previous study reported that lactate administration following 5–6 h of fasting decreased PDH^Ser293^ (i.e., PDH activation) in mouse skeletal muscle (Cerda-Kohler et al. 2018). However, we found that lactate administration did not affect PDH^Ser293^. In the current study, the protein content of PDK4, which inactivates PDH by phosphorylation, increased after overnight fasting (16 h) and acute exercise (1 h). Thus, we assume that the increased PDK4 protein content might result in no difference in the PDH phosphorylation state after lactate administration. Taken together, glucose uptake and oxidation does not appear to be associated with enhanced glycogen repletion in the plantaris and gastrocnemius muscles.

Other than enhanced glucose uptake and attenuated glucose oxidation, glyconeogenesis from lactate in skeletal muscle can contribute to glycogen accumulation. There had been an uncertainty and controversy regarding whether glycogen synthesis from lactate occurs in skeletal muscle, because some studies failed to detect the enzymes catalyzing the conversion of lactate to glycogen (Sacks & Sacks 1935; Eggleton & Evans 1930; Krebs & Woodford 1965). Based on these results, it was once concluded that lactate cannot be converted to glycogen in skeletal muscle, and that conversion of lactate to carbohydrate occurs only in the liver and kidneys (Krebs & Woodford 1965). However, subsequent studies demonstrated the presence of the lactate catalyzing enzymes in skeletal muscle (Opie & Newsholme 1967; McLane & Holloszy 1979; Crabtree et al. 1972). In addition, incorporation of lactate into glycogen in isolated skeletal muscle was confirmed using labeled lactate (McLane & Holloszy 1979; Shiota et al. 1984; Jin et al. 2015; Bonen et al. 1990), as first postulated in frog skeletal muscle (Hill 1924; Meyerhof 1925; Meyerhof 1920). Thus, it is feasible that lactate was incorporated into glycogen in the plantaris and gastrocnemius muscles.

Several studies have been undertaken to characterize the pathway by which lactate is converted to glycogen in skeletal muscle. The current understanding is that glyconeogenesis from lactate in mammalian skeletal muscle occurs mainly via the direct reversal of the pyruvate kinase reaction, permitting the formation of phosphoenolpyruvate from pyruvate, rather than via the malic enzyme route (Jin et al. 2015; Jin et al. 2009), and that muscular FBP, which catalyzes fructose-1,6-bisphosphate into fructose 6-phosphate, is the key regulatory enzyme for glycogen synthesis from lactate (McLane & Holloszy 1979; Shiota et al. 1984; Bonen et al. 1990; Pagliassotti & Donovan 1990; Xavier et al. 2002; Donovan & Pagliassotti 2000; Park et al. 2020). In mammals, there are two isotypes of FBP, liver FBP1 and muscle FBP2. The FBP1 protein mainly presents in the liver and kidneys, whereas FBP2 is predominantly expressed in skeletal muscle. In the present study, lactate administration did not increase glycogen content in the soleus muscle. In line with a previous finding that muscular FBP activity or protein content was substantially lower in oxidative muscle than in glycolytic muscle (Opie & Newsholme 1967; McLane & Holloszy 1979; Park et al. 2020), we observed significantly lower muscle FBP2 protein content in the soleus muscle than that in the plantaris and gastrocnemius muscles. These results suggest that oxidative muscles are less capable of synthesizing glycogen from lactate.

Contrary to muscular FBP, the protein content of MCT1, which mainly facilitates lactate influx from blood circulation into skeletal muscle, is inversely proportional to the percentage of fast-twitch fibers (Bonen et al. 2000). In agreement with this, the MCT1 protein content was significantly higher in the soleus muscle than in the plantaris and gastrocnemius muscles. Altogether, rather than lactate uptake at the sarcolemma membrane, glyconeogenic enzyme capacity appears to be the rate-limiting factor for glycogen synthesis from circulating lactate.

We should note the limitation that protein and glycogen contents assessed in the present study do not necessarily indicate definitive metabolic fluxes and glycogen synthesis from lactate. In addition, a previous study reported that there are considerable interspecies differences in the degree of glyconeogenic enzyme activities (Crabtree et al. 1972). While a previous study reported glyconeogenesis from lactate in rabbit skeletal muscle (Pagliassotti & Donovan 1990), others do not support direct glycogen synthesis from lactate in rat skeletal muscle (Azevedo Jr. et al., 1998). Although previous studies reported based on blood flow, arterial-venous difference, and gas analyses that lactate is converted to glycogen in human skeletal muscle (Hermansen & Vaage 1977; Astrand et al. 1986; Medbo et al. 2006; Nordheim & Vøllestad 1990; Bangsbo et al. 1991; Bangsbo et al. 1997), contribution of lactate to glycogen synthesis in human skeletal muscle is considered to be smaller than that in rodent skeletal muscle (Gleeson 1996; Gaesser & Brooks 1984). Moreover, to the best of our knowledge, no study using human muscle biopsy examined lactate incorporation into glycogen. Whether lactate directly contributes to post-exercise muscle glycogen restoration to a meaningful extent in humans should be examined in a future study.

## 5 Conclusions

In the present study, lactate administration and glucose ingestion additively enhanced post-exercise glycogen repletion in the plantaris and gastrocnemius muscles, but not in the soleus muscle. Nevertheless, lactate administration did not significantly change protein expressions related to glucose uptake and oxidation in the plantaris muscle, but it enhanced phosphorylation of TBC1D1, a distal protein regulating GLUT4 translocation, in the soleus muscle. We observed a content of the FBP2 protein, a rate-limiting enzyme that catalyzes lactate into glycogen, that was higher in the plantaris and gastrocnemius muscles than in the soleus muscle, and a content of MCT1 protein, which facilitates lactate uptake in skeletal muscle, that was higher in the soleus muscle than in the plantaris and gastrocnemius muscles. These results suggest that an elevated blood lactate concentration and post-exercise glucose ingestion additively enhance glycogen recovery in glycolytic phenotype muscles. This appears to be associated with glyconeogenic protein content, but not with enhanced glucose uptake, attenuated glucose oxidation, or lactate transport proteins.

## Funding

This research did not receive any specific grant from funding agencies in the public, commercial, or not-for-profit sectors.

## Author contribution statement

**Kenya Takahashi:** Conceptualization, Investigation, Data curation, Writing - original draft. **Yu Kitaoka:** Data curation, Writing - review & editing. **Yutaka Matsunaga:** Data curation, Writing - review & editing. **Hideo Hatta:** Conceptualization, Supervision, Writing - review & editing.

## Declaration of competing interest

The authors declare that they have no known competing financial interests or personal relationships that could have appeared to influence the work reported in this paper.
